# TOWARDS AN AGE-FRIENDLY UNIVERSITY: INSIGHTS FROM CULTURALLY DIVERSE COMMUNITY-DWELLING OLDER ADULTS

**DOI:** 10.1093/geroni/igae098.3908

**Published:** 2024-12-31

**Authors:** Joey Wong, Mario Gregorio, Paulina Santaella, Albin Soni, Jason Fu, Ilan Meghelli, Lillian Hung

**Affiliations:** University of British Columbia, Vancouver, British Columbia, Canada; University of British Columbia, Vancouver, British Columbia, Canada; University of British Columbia, Vancouver, British Columbia, Canada; University of British Columbia, Vancouver, British Columbia, Canada; University of British Columbia, Vancouver, British Columbia, Canada; University of British Columbia, Vancouver, British Columbia, Canada; University of British Columbia, Vancouver, British Columbia, Canada

## Abstract

Lifelong learning is essential for enhancing older adults’ self-esteem and social connections and expanding their capabilities. Including older adults on university campuses fosters intergenerational learning, where younger students benefit from their experiences and mentorship. This project addresses the growing emphasis on creating age-friendly universities by improving inclusiveness, diversity and equity at the University of British Columbia (UBC) Vancouver campus. The study, conducted in June and July 2024, engaged 25 community-dwelling older adults from diverse backgrounds—including Indigenous groups, multi-ethnic groups and people of color, LGBTQIA2+, and individuals with various physical and mental capacities—in audit walks with younger students across the campus. These walks assessed features related to “age-friendliness.” Group conversations before and after the walks provided valuable insights into participants’ perceptions of age-friendliness and feedback on current age-friendly university principles. Findings revealed a strong interest among older adults in continued learning, research collaboration, and mentorship opportunities. However, significant barriers were also identified regarding campus navigation, course registration, affordability, and communication. Additional concerns included safety issues, more accessible features, and improved transportation infrastructure. Participants also highlighted the importance of cultural inclusivity, such as multilingual resources and diverse art representations. This study not only reaffirms established age-friendly principles but also underscores the need to address environmental safety and accessibility from the perspectives of older adults. It advocates for a collaborative approach to developing an age-friendly university campus that effectively meets the needs of all its members, ensuring an inclusive environment for learners of all ages.

